# The Neuronal Responses to Repetitive Acoustic Pulses in Different Fields of the Auditory Cortex of Awake Rats

**DOI:** 10.1371/journal.pone.0064288

**Published:** 2013-05-16

**Authors:** Lanlan Ma, Xuhui Tai, Liye Su, Lijuan Shi, Enhua Wang, Ling Qin

**Affiliations:** 1 Department of Physiology, China Medical University, Shenyang, People's Republic of China; 2 Department of Otolaryngology,The No. 463rd Hospital of PLA, Shenyang, People's Republic of China; 3 Institute of Pathology and Pathophysiology, China Medical University, Shenyang, People's Republic of China; 4 Department of Physiology, Interdisciplinary Graduate School of Medicine and Engineering, University of Yamanashi, Chuo, Yamanashi, Japan; University of South Florida, United States of America

## Abstract

Cortical representation of time-varying features of acoustic signals is a fundamental issue of acoustic processing remaining unresolved. The rat is a widely used animal model for auditory cortical processing. Though some electrophysiological studies have investigated the neural responses to temporal repetitive sounds in the auditory cortex (AC) of rats, most of them were conducted under anesthetized condition. Recently, it has been shown that anesthesia could significantly alter the temporal patterns of neural response. For this reason, we systematically examined the single-unit neural responses to click-trains in the core region of rat AC under awake condition. Consistent with the reports on anesthetized rats, we confirmed the existence of characteristic tonotopic organizations, which were used to divide the AC into anterior auditory field (AAF), primary auditory cortex (A1) and posterior auditory field (PAF). We further found that the neuron's capability to synchronize to the temporal repetitive stimuli progressively decreased along the anterior-to-posterior direction of AC. The median of maximum synchronization rate was 64, 32 and 16 Hz in AAF, A1 and PAF, respectively. On the other hand, the percentage of neurons, which showed non-synchronized responses and could represent the stimulus repetition rate by the mean firing rate, increased from 7% in AAF and A1 to 20% in PAF. These results suggest that the temporal resolution of acoustic processing gradually increases from the anterior to posterior part of AC, and thus there may be a hierarchical stream along this direction of rat AC.

## Introduction

Both human speech and animal vocalizations are characterized by a low-frequency temporal modulation [1]. The representation of temporal information by auditory cortex (AC) neurons has been studied in various animal models and different animal preparations (for reviews see [2], [3]). The laboratory rat is a kind of important animal model for studies of auditory cortical function. Rat vocalizations exhibit modulations in 2–20 Hz range [4]. Some previous studies investigated the neural responses to temporal modulated sounds in the AC of rats. They found that the majority of AC neurons exhibited a sequence of transient discharges synchronized with the temporal envelope of sound stimulus, and the maximum repetition rate that can evoked the synchronous neural responses was generally lower than 20 Hz [5]–[7]. However, most of previous data were collected in the anesthetized rats. Recent studies of mammalian cortical processing are increasingly using awake preparations as they provide a more natural brain state than deep anesthesia. The effect of anesthetics on auditory cortical processing has been shown in several studies [8]–[11]. Therefore, it is necessary to examine the temporal processing of AC neurons in the awake rats.

It has been well established that the rat AC can be divided into several fields based on the properties of neural responses to pure-tone and noise [5]–[7], [12]–[15]. Similar to other animal models, such as the cat or nonhuman primate, the main criterion for functional characterization is tonotopy, which is a systemic spatial representation of tone frequencies driving the neurons. According to this, three different subfields have been most frequently reported in the core region of rat's AC: the primary auditory cortex (A1), the posterior auditory field (PAF) and the anterior auditory field (AAF). A1 was distinguished on the basis of an orderly tonotopic progression of from low to high frequencies along a posterior-to-anterior gradient. The tonotopic gradient reverses at the posterior and anterior borders of A1 to form boundaries with PAF and AAF, respectively. In addition, other features of the neuronal responses, such as their response latencies, intensity-response function and spectral tuning bandwidth, also differ among the neurons in relation to their position in the auditory fields.

Previous anesthetized experiments investigated the neural responses to tone/noise burst trains and sinusoid amplitude modulated (SAM) stimuli in A1 and PAF [5]–[7]. To date, only one study used the awake rats to examine the ability of multi-unit clusters in A1 in response to click-trains. The results showed that anesthesia could significantly alter the temporal patterns of neural response, and suppress the non-synchronized response [11]. The neural response of AAF to temporal modulated stimuli has not been investigated under either anesthetized or awake condition. For this reason, this study aims to cohesively examine the single-unit neural responses to click-trains across AAF, A1 and PAF, covering the core region of the rat's AC, under awake condition. Specifically, we evaluate whether the properties of neuronal responses to repetitive acoustic stimuli change along the rostral-to-caudal axis, aim to reveal how the temporal information of sound is processed among the AC. The results of the present study will provide an overall view of the temporal processing of the core region of rat AC in order to contribute to our understanding of the functional organization of the AC.

## Results

### BF organization of the AC of awake rat

**Figure 1:**
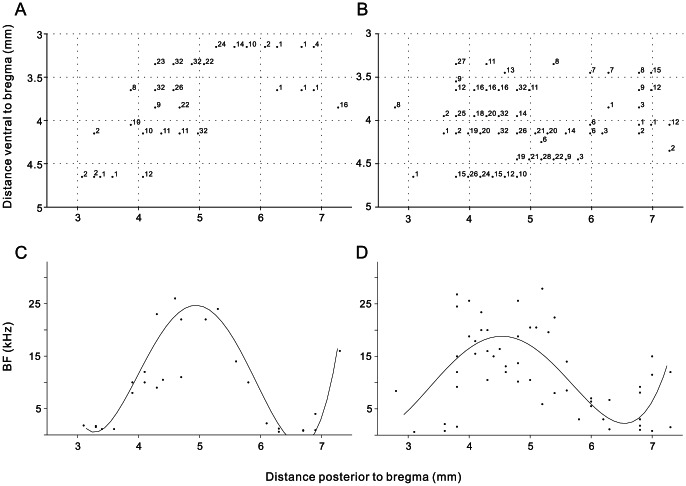
BF organization of rat AC in two animals. (A) and (B): scatter plot shows the coordinates of recording sites relative to bregma. Each number refers to the BF (in kHz) of a single-unit recorded in the middle cortical layers from an orthogonal penetration. (C) and (D): distribution of BF along the anterior-to-posterior direction obtained from the recordings sites shown in (A) and (B). Solid curve denotes the result of polynomial curve fitting of BF against distance posterior to bregma.

**Figure 2:**
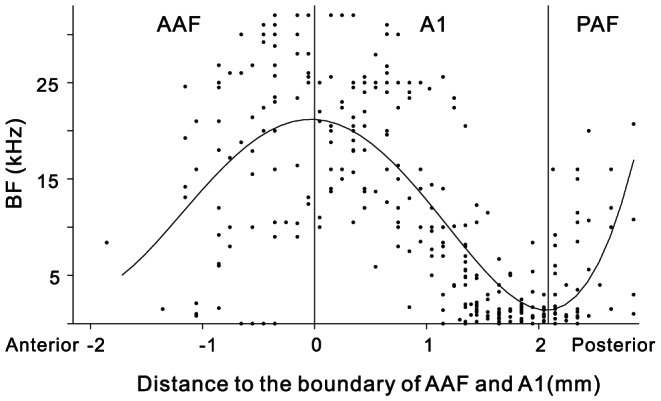
BF distribution of all data obtained from 12 animals. Conventions as Fig. 1C.

We conducted extracellular single-unit recording in left hemisphere of 12 awake rats. Daily recording sessions for each animal lasted 3–5 hours over 1–3 weeks. During the recording sessions, the animal stood fairly motionless some of the time and occasionally moved its limbs, whisked, groomed, etc. We successfully collected spike activities of 326 well isolated single units that showed a significant response to at least one of the click-trains tested. These units were recorded in cortical areas TE1 about 3–7 mm posterior of bregma and 3–5 mm lateral to bregma [5]. Consistent with the AC of anesthetized rats, an obvious characteristic of the AC of awake rats was also the BF gradient in the anterior-to-posterior direction. Figure 1A and 1B show coordinates of recording sites in the left AC from two example animals in which there were 31 and 57 penetrations, respectively. Number refers to the BF of each recording site. Figure 1C and 1D illustrate the spatial distribution of BF values in the anterior-to-posterior direction obtained from the recordings sites shown in Fig. 1A and 1B. Solid curve denotes the result of polynomial curve fitting of BF against distance posterior to bregma. A common feature is that a low-high gradient of BFs reversed to a high-low gradient at about 4.5 mm posterior to bregma. We coordinated the anterior-to-posterior distance of different animals at the location of BF reversal (estimated by the peak of BF fitting curve), and then overlapped the data of all 12 individual animals to construct a general scatter plot of BF distribution along anterior-to-posterior axis (Fig. 2). More clearly, as the recording site moves from the anterior to posterior side, BF firstly increases from low to high frequency, then decreases from high to low frequency, and reverses to high frequency again at the posterior end. According to the reports of anesthetized rats, the reversals of BF gradient could be used to define the boundaries between AAF, A1 and PAF [5]–[7], [12]–[15]. Using this method, we divided 86 units of our data into AAF, 140 units into A1 and the remaining 100 units into PAF.

### Representative examples of neural responses to click-trains

**Figure 3:**
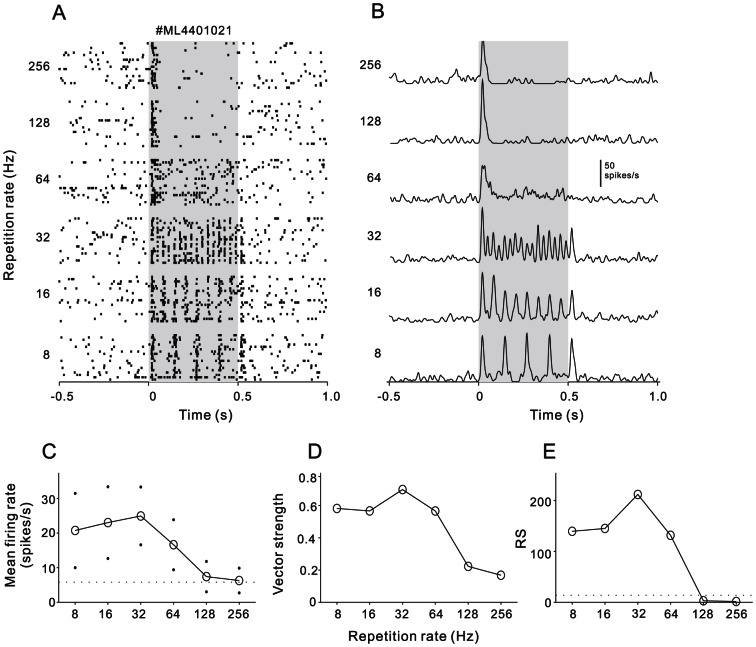
An example neuron showing stimulus-synchronized responses. (A) Raster plots of neural spikes in response to 20 repetitions of 8–256 Hz click-trains. Each dot represents one action potential. Shaded gray region shows the stimulus duration. (B) PSTH representing the firing rate averaged across the 20 trials of each stimulus condition (1-ms bin, smoothed by Gaussian function with 5-ms SD). (C) Mean firing rate as a function of the repetition rate. Dots indicate the SD. Dashed horizontal line shows the background firing rate. (D) Vector strength as a function of the repetition rate. (E) Rayleigh statistic for neuronal response shown in (A). Responses to 8–64 Hz click-trains beyond the criteria for statistical significance (13.8, P<0.001) which is indicated on the plot with a dashed horizontal line.

The cortical neurons of the awake rat showed different firing patterns in response to the click-train stimuli. We firstly present several representative examples of the neural responses. Figure 3A and 3B show a raster plot and PSTH from one AAF neuron. At 8 Hz of repetition rate, the timings of clicks were explicitly represented by the timings of the spikes. Such a phase locked response pattern remained when the repetition rate increased to 64 Hz, but the number of spikes locked to the later clicks gradually decreased. Specially, at the repetition rate of 64 Hz, the neural response of individual stimulus trial could not continuously follow the click-train; only the mean PSTH averaged over 10 trials displays a fine fluctuation reflecting the stimulus repetition rate, suggesting that 64 Hz is the limitation of this neuron to follow. At the repetition rates>64 Hz, the neural response soon adapted to the spontaneous level after the stimulus onset, could not lock to the following clicks. Consequently, the mean firing rate driven by the entire click-trains also deceased with the increase of stimulus repetition rate (Fig.3C). We used the vector strength (VS) to measure the degree of the neural response to synchronize to the click-trains (See Materials and Methods). Figure 3D displays the function of VS against the repetition rate. VS peaked at 32 Hz, which was defined as the best synchronization rate. Rayleigh Statistic (RS) was used to evaluate statistical significance of the spike synchronization to clicks (Fig. 3E). RS was higher than 13.8 (corresponding to p<0.001 in the Rayleigh test) at 8–64 Hz, and lower than 13.8 at 128–256 Hz. Therefore, the synchrony boundary of this neuron was 64 Hz. That means the temporal information repeated lowered than 64 Hz could be well represented by the spike-time of this neuron.

**Figure 4:**
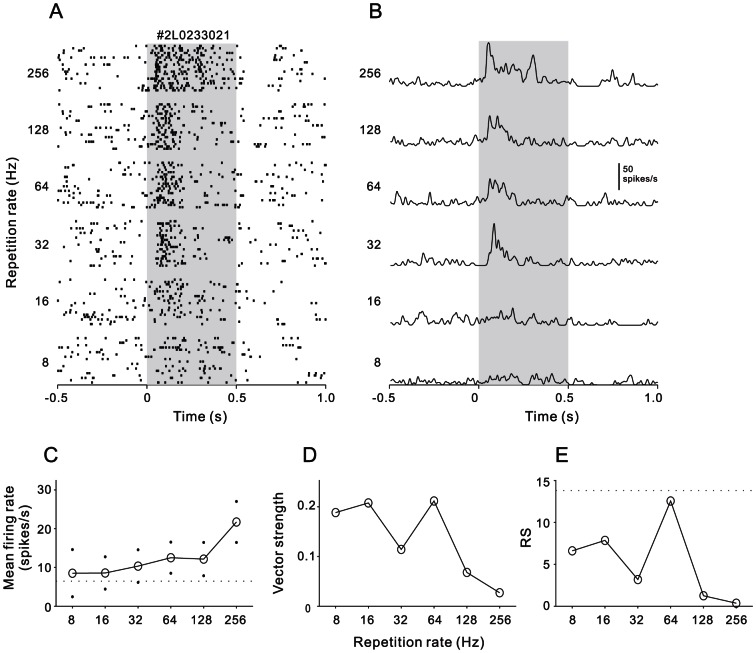
An example of non-synchronized neuron showing a rate-coding of repetition rate. Conventions as Fig. 1

A study on awake marmoset had reported that a subpopulation of AC neurons showed a non-synchronized response continuing throughout the stimulus period [16]. Such a sustained response pattern was also found in our sample of awake rat. Figure 4 presents a neuron recorded from PAF. The mean firing rate of this neuron monotonically increased with the increase of stimulus repetition rate (Fig. 4C). On the other hand, VS of this neuron fluctuated at low levels (Fig. 4D), and RS was below 13.8 at any repetition rates (Fig. 4E). Thus, this neuron did not synchronize to the click-train stimuli, and only used the mean firing rate to represent the stimulus repetition rate.

**Figure 5:**
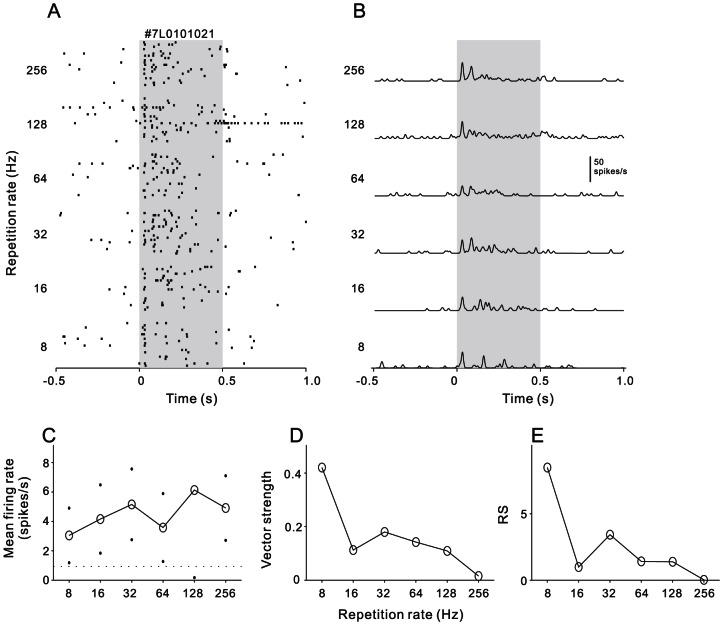
An example neurons showing non-synchronized response to all the click trains, but the firing rate was not significantly modulated by repetition rate. Conventions as Fig. 1.

There were also some neurons that significantly elicited by the click-trains, but did not synchronize to any of the stimuli and its mean firing rate was not significantly modulated by the repetition rate (ANOVA, p>0.05; Fig.5). Thus, neither the spike-time nor firing rate of this kind of neuron can effectively represent repetition rate.

### Population neural responses to click-trains

**Figure 6:**
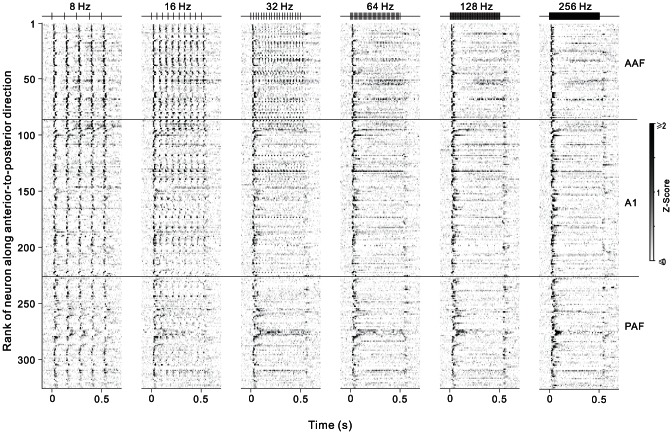
Individual PSTHs depicting the response patterns of 326 neurons to different click-trains. The absolute firing rates of each neuron were normalized to Z-scores and, for visualization purposes, smoothed and displayed in grayscale plots. PSTHs are aligned at the onset of stimulus. Within each plot, neurons were ranked by the relative location in the anterior-to-posterior direction. Horizontal lines mark the boundaries between AAF, A1 and PAF. Stimulus envelopes are shown at the top of each plot.

To illustrate the response patterns of the neuron population, we present a stack of Z-score PSTHs of the 6 click-trains at different repetition rates for the 326 recorded neurons in Fig. 6. For visualization purpose, the PSTH of each neuron was assessed by the normalized value of the Z-score, which was calculated by subtracting the mean background firing rate (averaged across the all trials of 500 ms pre-stimulus period) from the firing rate, and then divided by the SD of the background firing rate. White in the plot indicates lower than the background firing rate. Black indicates firing that is≥2 SD of the background firing rate. In each panel, the PSTHs from different neurons are arranged in ascending order of the distance posterior to bregma. Horizontal lines mark the boundaries between AAF, A1 and PAF. A salient feature of the population PSTHs is that the majority of AAF neurons showed a serial of transient responses clearly synchronized to the 8 Hz click-train, and the stimulus-synchronized response gradually deteriorated as the recording site moved to the posterior side of AC. And with increasing the stimulus repetition rate to 16–64 Hz, the synchronized response progressively degraded from the posterior side of AC. At the repetition rate of 128–256 Hz, the dominant response pattern across all the AC neurons became to a phasic response occurred only at the stimulus onset. A part of neurons also showed sustained response during the stimulus period or phasic response at the stimulus offset. These results indicate that there is a clear descending gradient of stimulus-synchronizing ability in the anterior-to-posterior direction of AC, and the highest repetition rate for the AC to follow is about 64 Hz.

### Quantitative comparison of the neural responses in AAF, A1 and PAF populations

**Figure 7:**
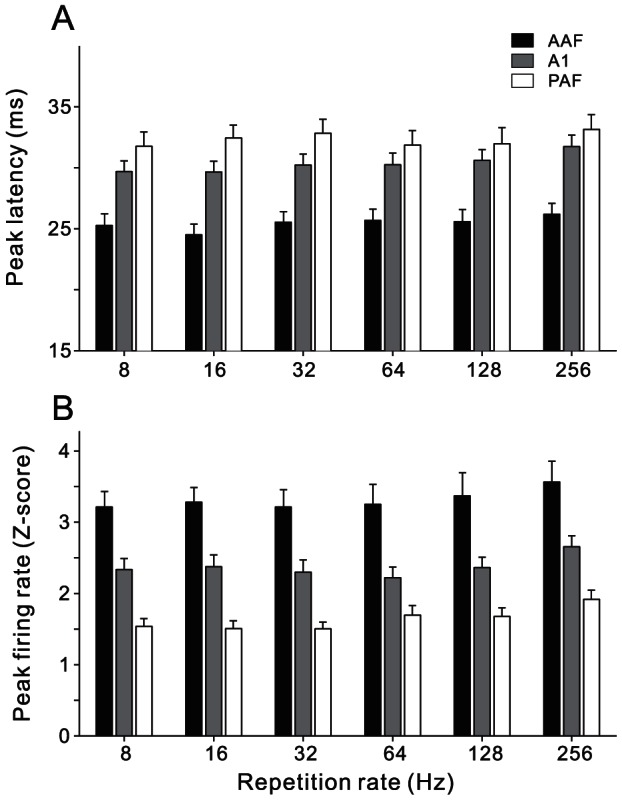
Bar plots comparing peak latency (A) and response amplitude (B) at 8–256 repetition rates for neurons in AAF, A1 and PAF. Error bars indicate S.E.

To quantitatively compare differences in the neural responses among different auditory cortical fields, we firstly measured the peak latency of each unit, the time that the PSTH reached the first response peak. Figure 7A displays the mean and S.E. (standard error) of peak latency in the neurons of AAF, A1 and PAF. For all the tested repetition rates (8–256 Hz), the mean peak latency of AAF neurons was fastest (25 ms), that of PAF neurons was slowest (33 ms), and that of A1 neurons was intermediate (30 ms). The difference among the three neuron groups was statistically significant in all the 6 stimulus conditions (ANOVA, p<0.05). In addition, the peak amplitude of neural response was also significantly different among the cortical fields (Fig. 7B). AAF neurons showed the strongest response; PAF neurons showed the weakest response; and A1 neurons were intermediate (ANOVA, p<0.05, for all the 6 stimulus conditions). Therefore, the anterior part of AC was elicited faster and stronger by the click-trains than the posterior part of AC.

**Figure 8:**
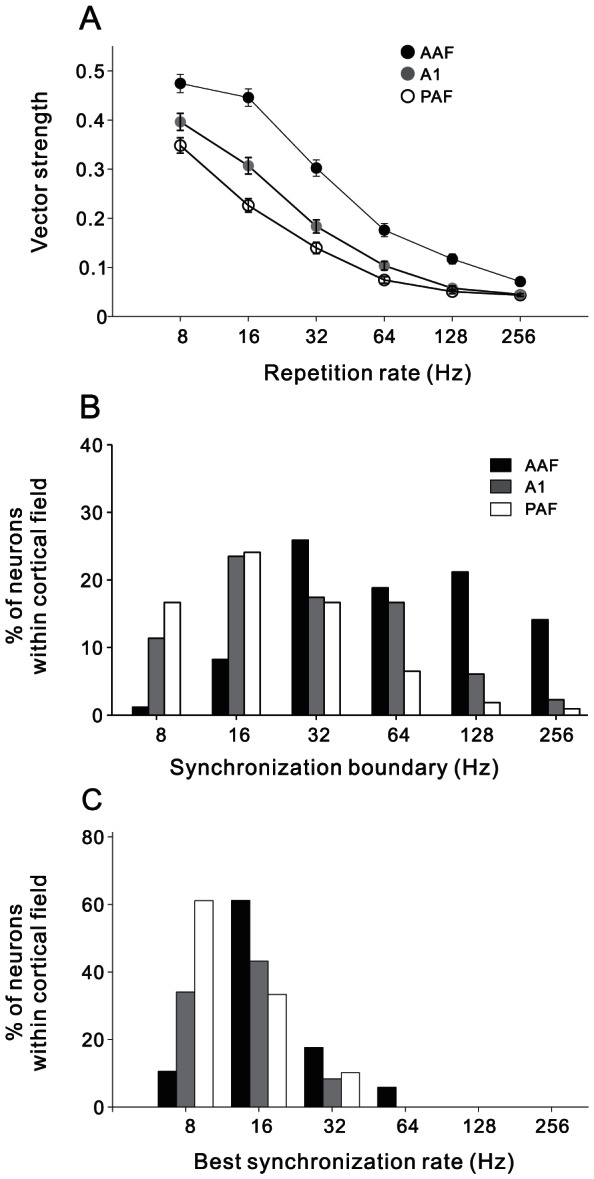
Stimulus-synchronization capability of AAF, A1 and PAF populations. (A) mean VS across the population of AAF, A1 and PAF neurons against stimulus repetition rate. Error bar indicates the S.E. (B) distribution of synchronization boundaries among each cortical field. (C) distribution of best synchronization rate among each cortical field.

We then quantitatively compared the stimulus-synchronization ability between the 3 cortical fields. Figure 8A shows the mean function of VS against stimulus repetition rate in AAF, A1 and PAF neurons. All the 3 functions decreased with the increase of repetition rate, while at all repetition rates, the mean VS was the highest in AAF, and the lowest in PAF. In Fig. 8B, we compared the distributions of synchronization boundary (the maximum repetition rate at which significantly synchronized response was observed) in AAF, A1 and PAF neurons. The median of synchronization boundary was 64, 32 and 16 Hz in AAF, A1 and PAF, respectively. The difference between the synchronization boundaries was statistically significant (Kruskal-Wallis test, p<0.01). The distributions of best synchronization rate were also significantly different (Kruskal-Wallis test, p<0.01, Fig. 8C). Both the median of AAF and A1 neurons was 16 Hz, while that of PAF neurons was 8 Hz. All above analysis confirmed the visual inspection of Fig. 6 that AAF neurons have the highest ability to follow the repeated stimuli, PAF have the lowest ability, and A1 are intermediate. Therefore, the stimulus repetition rate is more precisely encoded by the spike-time of neurons in the anterior part of AC than that in the posterior part.

### Compositions of synchronized and non-synchronized neurons in AAF, A1 and PAF

**Figure 9:**
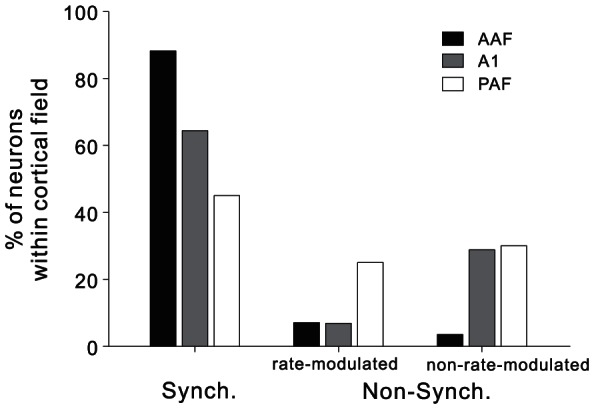
Comparison of the proportion of different types of neurons in AAF, A1 and PAF.

As illustrated by the examples in Figs. 4 and 5, some neurons did not exhibit a significant synchronous response to any of the test click-trains. We designated these neurons as non-synchronized neurons. The non-synchronized neurons could be further divided into rate-modulated and non-rate-modulated types, according to whether the mean firing rates of different stimulus groups were significantly different (ANOVA, p<0.05) or not. The proportion of different types of neurons in AAF, A1 and PAF is displayed in Fig. 9. In AAF, only 12% of neurons were non-synchronized neuron, and 7% was rate-modulated, and 5% was non-rate-modulated. In A1, the proportion of non-synchronized neurons increased to 36%, in which the proportion of rate-modulated neurons unchanged (7%), while that of non-rate-modulated neurons substantially increased (29%). In PAF, the proportion of non-synchronized neurons further increased to 56%, this was mainly due to a significant increase in the proportion of the rate-modulated neurons (26%). Therefore, although the number of neurons synchronizing to the stimuli decreased in PAF, more neurons could use the variety of mean firing rate to encode the repetition rate. This result was consistent with the study on awake marmosets reporting that there was a transformation from temporal to rate coding between primary and non-primary auditory cortical areas [17].

## Discussion

In the present study, for the first time, we systematically examined the single-unit neural responses to a series of click trains in the core region of the rat AC under awake condition. Consistent with the studies of anesthetized rats, we confirmed the existence of characteristic BF gradients along the anterior-to-posterior direction of AC, which can be used to divide the core region of AC into AAF, A1 and PAF (Figs. 1 and 2). Our present study further present an overall view of neural response to click-trains of AC, revealed a gradual decrease of stimulus-synchronized response along the anterior to posterior direction. In addition, more non-synchronized neurons, whose mean firing rate was modulated by the repetition rate, appeared in the posterior AC. The results of the present study provide an overall view of the temporal processing of the core region of rat AC and thus contribute to our understanding of the functional organization of the AC.

### Comparison of the stimulus-synchronization responses from anesthetized and awake animals

The ability of cortical neurons following to the repetitive elements of an auditory stimulus has been well investigated for a long time using different animal species, anesthetic state, and analytical methods. To date, the most part of data was collected on anesthetized animals. Studies using anesthetized cats have found the limiting rate followed by A1 neurons was typically below 25 Hz [3], [18]–[20]. In anesthetized macaque monkeys, synchronized responses were most frequently observed at click rates below<12 Hz, the median of limiting rate of A1 neurons in was 25 Hz [21]. In the rat, a previous study using sinusoidal amplitude modulated (SAM) sounds reported that neurons typically responded best to SAM rates of 2–10 Hz and were generally unresponsive to SAM rates above 20 Hz [5]. Similar best and limiting rates of neural responses were also observed when the rats were stimulated by tone and noise trains [6], [7]. Therefore, the majority of synchronization boundaries were about 20 Hz for the auditory cortical neurons of various species.

However, several studies on awake animals have shown that the synchronization ability of cortical neurons in awake state was higher than that in anesthetized state. Stimulus-synchronized multi-unit activity in the AC of awake macaque monkeys occurred up to 300 Hz using click trains [22]. The median synchronization boundary for single-unit activity in the AC of awake marmosets was about 50 Hz [16]. Recordings from unanesthetized cat auditory cortex revealed the median of limiting rate was between 50–100 Hz [23]. The mean of synchronization boundary for multi-units in the A1 of awake rats was 71 Hz using vector strength measurement [24].

Previous awake studies seldom systemically compared the synchronization ability of neurons between multiple cortical fields in the same animal. Our present study showed that the synchronization boundaries were different between the subfields of rat AC. The median value was 64, 32 and 16 Hz in AAF, A1 and PAF, respectively (Fig. 8B). It is difficult to compare the synchronization boundaries of our data directly with those of previous awake experiments, because there were many differences in the experimental conditions, such as animal species, definition of cortical subfields, and electrical recording method. Nevertheless, our result is consistent with the general tendency that neurons in awake state had higher synchronization ability than in anesthetized state.

### Non-synchronized responses

The study on awake marmosets also reported that about half of auditory cortical neurons exhibited a non-synchronized response lasting the entire duration of the stimulus train [16]. The mean firing rate of these neurons can encode the stimulus repetition rate. The non-synchronized response pattern was more frequently observed at the stimulus repetition rate>40 Hz, at which most of neurons cannot follow the stimuli. Therefore, the complementary activities of synchronized and non-synchronized neurons may constitute a two-stage mechanism to represent a wide range of repetition rates. The timing of relatively slowly occurring acoustic events can be explicitly represented by the temporal discharge patterns of the stimulus-synchronized neurons. Faster stimulus rates can be implicitly represented by the average discharge rate of non-synchronized neurons. In the present study, we found two forms of non-synchronized response in awake rats: one was that the mean firing rate was significantly modulated by the stimulus repetition rate, which may present some encoding information of repetition rate (Fig.4); the other was that the mean firing rate was less modulated by the repletion rate (Fig. 5). The proportions of both kinds of non-synchronized responses were very low in AAF (7% and 5%). In A1, the large majority of non-synchronized responses were non-rate-modulated. There were only 7% of the non-synchronized responses encoding the repetition rate. This result was consistent with the multi-unit studies on A1 of awake rats in which 8–10% of recorded clusters exhibited a similar rate coding pattern [11], [24]. Other two studies on awake macaque monkeys also reported that only a small proportion of A1 neurons (2% and 17%) showed the exclusively non-synchronized responses [25], [26].

However, in PAF, the percentage of neurons using firing rate to encode repetition rate increased to 26% (Fig. 9). Meanwhile, the percentage of PAF neurons synchronized to the stimuli substantially decreased comparing to AAF and A1. Therefore, the spike-time coding of repetition rate gradually degrades as the processing stream moves along the anterior-to-posterior direction of AC. In the posterior AC, rate coding plays a more important role in the representation of repetition rate. This tendency supports the proposal that there is a conversion from temporal to rate code in non-primary auditory cortical fields [17]. In spite of this, the total percentage of rate-modulated non-synchronized response in our awake rats was smaller than that reported on awake marmosets. Except for the species difference, different sound stimuli used in our and Lu et al. studies may also account for the disparity [16]. To collect more units in limited recording time, we applied the same set of wide-band click-trains (composed by a series of rectangle pulses) to stimulate all the recorded neurons, while Lu et al. used the narrow-band click-trains, in which the spectral peak and bandwidth was optimally chosen on a neuron-by-neuron basis. It has been shown that sustained response was more frequently found when the auditory cortex neurons were stimulated by optimized stimuli [27].

### Acoustic processing in AAF, A1 and PAF

On the basis of thalamocortical projections, the AC of rat is anatomically subdivided into a central core region designated as TE1 and surrounding belt regions labeled TE2 and TE3[28]. The A1, AAF and PAF occupy the core region of rat AC [5], [13]. Our data confirmed the previously described differentiation of the core region of AC based on its tonotopicity into 3 fields: AAF, A1 and PAF [5]–[7], [12]–[15]. There is a clear tonotopic gradient in the A1 with low BFs in the anterior part and high BFs in the posterior part. The reversal of tonotopic gradient in the high BF side marks the border of A1 and AAF. The reversal of tonotopy in the low BF side of A1 demarcates PAF. We also found that the response latency was shorter and the response amplitude was stronger in the anterior than in the posterior AC. Analysis of neural response latency has been useful in developing models of visual cortical function [29], [30]. Recently, neural latency was also used to reveal the auditory processing stream in cats [31] and primates [32]. In anesthetized rats, some previous studies reported that the mean onset latency was typically longer in PAF than in A1 [5], [7], and others reported that AAF neurons exhibit shorter response latencies than A1 neurons [13]. Our results from the same rat under awake condition confirmed that response latency increases in a sequence of AAF, A1 and PAF, suggesting that there may be a hierarchical auditory processing directed from AAF to PAF. Similar acoustic information flow has also been suggested in cat AC [31].

It has been proposed that there is a progressive reduction in the maximum following rate at ascending levels of the auditory neuraxis [2], [3], [33]. In cats, the maximum following rate in response to temporally modulated stimuli of neurons in PAF is considerably slower than neurons in A1 and AAF [18]. Previous studies on anesthetized rats only compared the temporal responses between A1 and PAF neurons and found that the limiting rate of PAF was significantly slower and response synchronization was reduced compared with A1 [7]. Our results showed a progressive degradation of synchronization ability among AAF, A1 and PAF neurons, further suggesting a hierarchical elation between the 3 cortical fields. Collectively, these results demonstrate that despite the substantial reduction in physical scale, the rat AC shares an organization similar as other well-described AC models.

## Conclusion

The rat has been widely used to investigate the auditory cortical processing and plasticity. However, compared to other animals, there have been relatively few studies investigating its neurophysiological properties under awake condition. Our results confirm and extend these previous neurophysiological studies and additionally have the advantage of measuring neural response properties across a large extent of the AC in the same rat under awake condition. We found that BF exhibits characteristic tonotopic organizations in the core region of AC, similar to those in anesthetized rats. AC neurons in awake state show higher capability to follow temporal repetitive stimuli than in anesthetized state. The neuron's maximum following rate progressively decreases along the anterior-to-posterior direction of AC, while more neurons in the posterior part of AC show non-synchronized responses to repetitive stimuli and can use mean firing rate to represent the stimulus repetition rate. These results suggest that the temporal resolution of acoustic processing gradually increases from the anterior AC to the posterior AC, and thus there may be a hierarchical stream along this direction of rat AC.

## Materials and Methods

All animal works was carried out in strict accordance with the recommendations in the Guide for the Care and Use of Laboratory Animals of the National Institutes of Health. The protocol for animal handling and the treatment procedures were reviewed and approved by the China Medical University Animal Care and Use Committee (permit number of CMU62043018). All surgery was performed under anesthesia, and all efforts were made to minimize suffering.

### Surgical preparation, recording procedure and histology

Animals were anaesthetized with 10% chloral hydrate (0.33 ml/100 g), and then fixed on the stereotaxic frame (SR-5R, Narishige, Tokyo, Japan). Temperature was monitored rectally and maintained at 37°C using a feedback-controlled blanket. Supplemental dosages of chloral hydrate were provided when required. The cranium was exposed, four small holes were drilled over the parietal bone and fine jeweler's screws were inserted to serve as an anchor for a metal head-post holder that was cemented to the skull with dental acrylic. Stainless-steel wires were soldered onto two screws as a ground. The location corresponding to the core region of AC (TE1)[28], was marked on the surface of temporal bone according to the coordinates of the Paxinos and Watson brain atlas: 3–7 mm posterior of bregma and 3–5 mm lateral to bregma [34]. A plastic chamber with removable screw cap was implanted on the temporal bone over the marked area for microelectrode access. A short length of 27-gage stainless-steel tubing was embedded into the acrylic cement near the bregma to serve as a reference pin during chronic recording. After surgery, an antibiotic (Cefuoxime, Zinacef®njection, Glaxosmithkline) was administered systematically (30 mg/kg, i.p.).

The rat was allowed at least 24 h of recovery time before the first recording session. All recording sessions were conducted within a shielded, soundproof room. During the recording session, the head was fixed through the head-post holder and the animal was standing inside a half-cut plastic tube (diameter, 5 cm), which provided a loose restraint for body movements. The cap of plastic cylinder was removed. A small hole (diameter, 0.5–1 mm) was drilled in the skull, and the dura was pierced with a sharpened probe, and then a single epoxylite-insulated tungsten microelectrode (FHC Inc.; impedance: 2–5 MΩ at 1 kHz) was inserted stepwise with a pulse motor-driven manipulator (SM-20, Narishige, Tokyo, Japan). The electrode penetration, perpendicular to the cortical surface, was made under visual guidance via an operating microscope. This gave good control and estimates of recording location and depth. To obtain more units from different areas during the limited time of awake experiment, the electrophysiological recordings were focused on the depth of approximately 400–600 µm from the pial surface, corresponding to the thalamorecipient layers (layer III/IV) of rat AC. Tucker–avis Technologies (Alachua, FL) neurophysiology hardware (RA16PA, RZ-2) and software (OpenEx) were used for signal filtering (0.3–5 kHz), amplification, data acquisition. Action potentials were detected on-line by threshold crossing, and waveforms were stored to hard disk. Single-unit spike activities were sorted off-line using a principal component analysis (OpenSorter, TDT, Alachua, FL).

At the end of each recording session, the recording hole was thoroughly rinsed with sterile saline and filled with an antibiotic ointment. The plastic chamber was then filled with a polyvinylsiloxane dental impression material that provided a tight seal and could be removed easily at the next recording session. After 2–5 electrode penetrations were made within one recording hole, the hole was sealed by dental cement and a new hole was opened. Electrode penetration points were coordinated to the implanted reference pin and marked on a digitized photograph of the bone surface.

Daily recording sessions for each animal lasted 3–5 hours over 1–3 weeks. A video camera was placed in front of the animal to monitor its state. During the recording sessions, the animal stood fairly motionless some of the time and occasionally moved its limbs, whisked, groomed, etc. Any recording data interrupted by the artifacts of the animal's movement were abandoned, and the recordings were repeated as the animal returned to resting state. On average, 1–3 well-isolated single-units were collected in each daily session.

In the end of all recording experiments, some electrode tracks were marked with electrolytic lesions. The animal was then transcardially perfused under deep anesthesia. The brain was cut in coronal sections and stained with neutral red. The location map of recording sites was constructed on a digitized photograph of the auditory cortex surface by calibrating the coordinates of the lesion sites.

### Acoustic stimuli

Acoustic stimuli were digitally generated by custom-built programs under MATLAB (Mathworks) environment and delivered via a free-field speaker (K701; AKG). The speaker was placed at the horizontal plane 1 m from the pinna and 45° contralateral to the recording hemisphere. Frequency and intensity calibrations were done with a Bruel & Kjaer 1/2″condenser microphone with a preamplifier 2669 situated at the rat's ear. Sound pressure level (SPL) is expressed in decibels relative to 20 µPa. Click-trains were composed of monopolar, rectangular pulse of 0.2 ms duration at the repetition rates of 8, 16, 32, 64, 128 and 256 Hz for 0.5 s. The click-trains at different repetition rates were randomly interleaved and repeated 20 times with inter-train-intervals of>1 s. Stimulus amplitudes were adjusted to the subjective intensity equal to 55 dB SPL of 4 kHz pure tone. No loud stimuli were applied to avoid refectory pinna movements (Preyer reflex). A set of 125 pure-tone stimuli (160 ms duration and 55 dB SPL, ranging from 0.2 to 32 kHz) was presented prior to the click-train stimuli to examine the best frequency (BF, the frequency evoked the largest number of spikes) of each unit.

### Data analysis

Spike activities driven by click-trains were aligned along the stimulus onset, constructing a raster plot of each repetition rate (Fig. 3A). The peri-stimulus time histogram (PSTH), counting the spikes across different repetition rates, was computed in 1-ms bin width (Fig. 3B, for visualization purpose, the PSTH was smoothed by Gaussian function with 5 ms SD). Peak latency was quantitatively determined as the time to reach the first peak response in the PSTH, and the maximum peak height of the PSTH was designated as peak firing rate. The spontaneous firing rate for 500 ms before stimulus onset was considered as background. The mean + 2SD of background firing rates across the trials of all stimuli was deemed as the threshold level to identify a significant response. A neuron was considered to significantly respond to a click-train, if its peak firing rate was higher than the threshold level. When a neuron was found to significantly respond to at least one of the click-train stimuli, we calculated mean firing rate over the duration of click-train stimulus plus 100 ms following the stimulus, and plotted the mean firing rate against the repetition rate of click-trains (Fig. 3C). A neuron was considered rate-modulated if ANOVA test revealed a significant difference among the neuron's mean firing rates from the six stimulus groups (p<0.05).

Vector strength (VS)[35] was used to measure the degree to which the neural response was concentrated in a particular phase of the repetition period of the clicks, such that 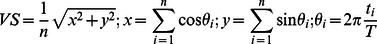 where n is the total number of spikes, t_i_ is the time of spike occurrence, and T is the inter-click interval. VS values ranged from zero (spikes evenly distributed throughout the stimulus period) to one (spikes are perfectly aligned to a particular phase of the stimulus period). The VS of each neuron was calculated over the time period starting from 50 ms after stimulus onset to 50 ms after stimulus offset. VS was plotted against the repetition rate of click-trains (Fig. 3D). A neural response was considered to be synchronized to the click if its VS was greater than 0.1 and the Rayleigh statistic (RS), 2nVS^2^, exceeded 13.8 (p<0.001)[36]. The highest repetition rate at which VS was significant was identified as ‘synchronization boundary’ [16]. The best synchronization rate was defined as the repetition rate at which the VS reached its maximum.
